# Supplementary material for: Mechanism of lateral cell-wall expansion at a constant diameter in Bacillus subtilis
Source: Nat Commun. 2025 Jul 19;16:6671. doi: 10.1038/s41467-025-61900-0 (PMC12276332; doi:10.1038/s41467-025-61900-0)
Supplement: Supplementary file 1 — Supplementary information [file 41467_2025_61900_MOESM1_ESM.pdf]

Mechanism of lateral cell-wall expansion at a constant diameter in *Bacillus subtilis*

Yucheng Liang[1], Laure Bellard[2], Yung-Sing Wong[3], Cécile Morlot[2], Jean-Emmanuel Hugonnet[1*], Filippo Rusconi[1,4,a,*], and Michel Arthur[1,a,*]

[1] *INSERM ERL 1336, UMR 8228, Sorbonne Université-ENS-PSL-CNRS, Paris, F-75006, France*

[2] *Univ. Grenoble Alpes, CNRS, CEA, IBS, 38000 Grenoble, France.*

[3] *Univ. Grenoble Alpes, CNRS, DPM, 38000 Grenoble, France.*

[4] *GQE-Le Moulon/PAPPSO, Université Paris-Saclay, INRAE, CNRS, AgroParisTech, IDEEV; 12, route 128; F-91272 Gif-sur-Yvette, France.*

[*]Corresponding authors
Jean-Emmanuel Hugonnet: jean-emmanuel.hugonnet@crc.jussieu.fr
Filippo Rusconi: filippo.rusconi@universite-paris-saclay.fr
Michel Arthur: michel.arthur@crc.jussieu.fr

[a] Equal contribution

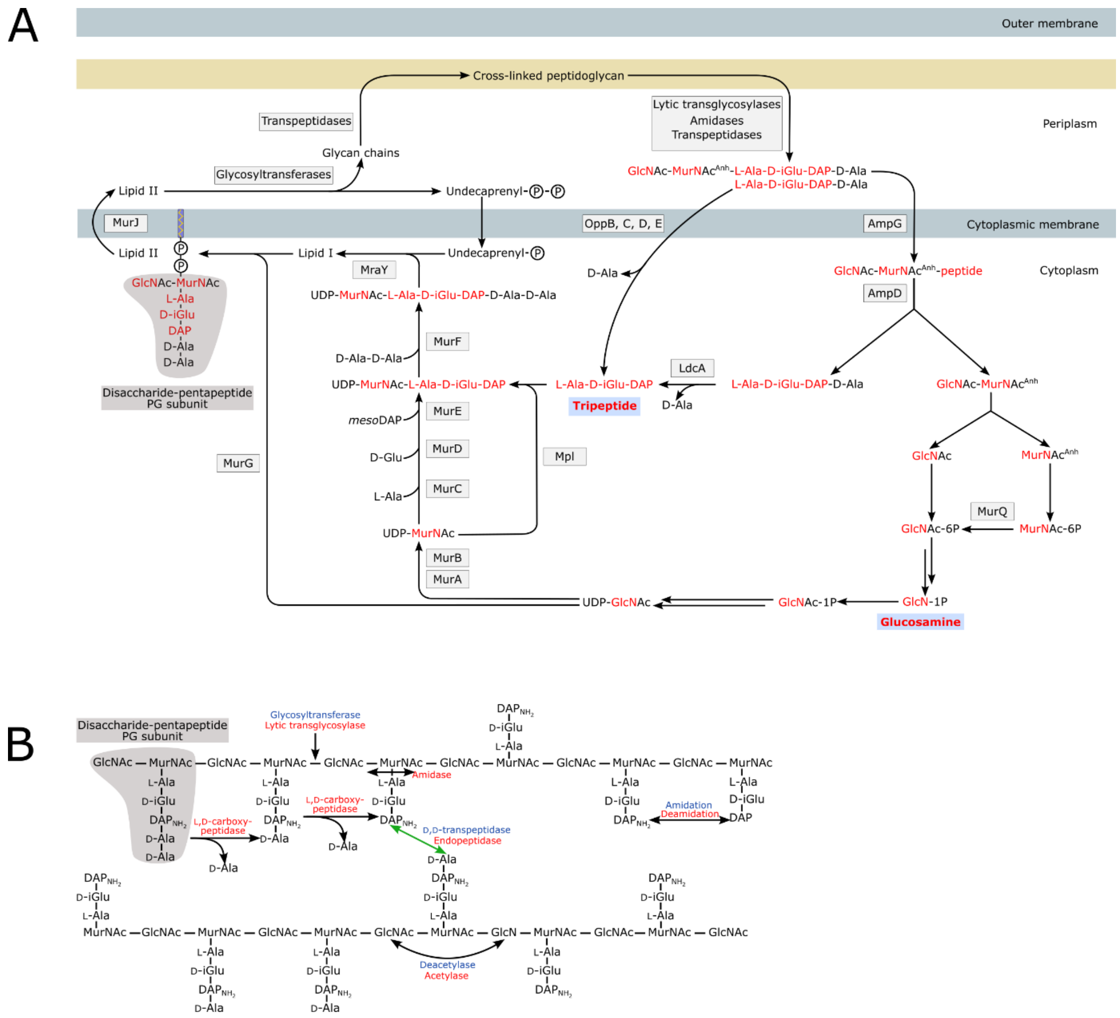

Supplementary Fig 1. Peptidoglycan assembly and maturation reactions.

A. PG biosynthesis and recycling pathways in *Escherichia coli*. Synthesis of the disaccharide-pentapeptide subunit involves the production of UDP-MurNAc from UDP-GlcNAc by MurA and MurB, followed by sequential addition of L-Ala, D-Glu, and *meso*DAP to form UDP-MurNAc-L-Ala-D-iGlu-DAP. MurF then adds the D-Ala-D-Ala dipeptide to form UDP-MurNAc-pentapeptide. The phospho-MurNAc-pentapeptide moiety of this precursor is transferred to the lipid carrier (undecaprenyl-phosphate; blue rectangle in the cytoplasmic membrane), followed by the addition of GlcNAc. These reactions yield the complete disaccharide-pentapeptide subunit (highlighted in grey) linked to the undecaprenyl-lipid carrier via a pyrophosphate bond. The disaccharide-pentapeptide subunit is then translocated to the cell surface by the MurJ flippase and polymerized into glycan chains by glycosyltransferases. PG biosynthesis is completed by transpeptidases that form the cross-links connecting adjacent glycan chains. PG recycling is initiated by the cleavage of the PG mesh by lytic transglycosylases, amidases, and endopeptidases, thus generating peptides and GlcNAc-MurNAc^{Anh}-peptide fragments, which are transported into the cytoplasm by the Opp and AmpG permeases, respectively. Two PG moieties are recycled: (i) the tripeptide L-Ala-D-iGlu-DAP, which is added to UDP-MurNAc by the dedicated recycling enzyme Mpl and (ii) the glucosamine (GlcN) moiety of GlcNAc and MurNAc, which is recycled in the neosynthesis of both of these sugars. Of note, the acetyl group (Ac) of GlcNAc and MurNAc and the D-lactoyl groups of MurNAc do not originate from recycling, the latter being indicated by partial red coloring of the residue name.

B. Periplasmic PG biosynthesis and maturation steps in *Bacillus subtilis*. Enzymes involved in biosynthetic and maturation reactions are shown in blue and red, respectively. The PG is assembled from a disaccharide-pentapeptide subunit (highlighted in grey). The green double arrow indicates a cross-link between stem peptides. The black double arrow indicates the bond cleaved by amidases. Glycosyltransferases polymerize glycan strands by forming β-1→4 glycosidic bonds. Lytic glycosyltransferases cleave the MurNAc-GlcNAc bond and form an internal anhydro bond, producing MurNAc^{Anh}. DAP_{NH2}, amidated diaminopimelic acid.

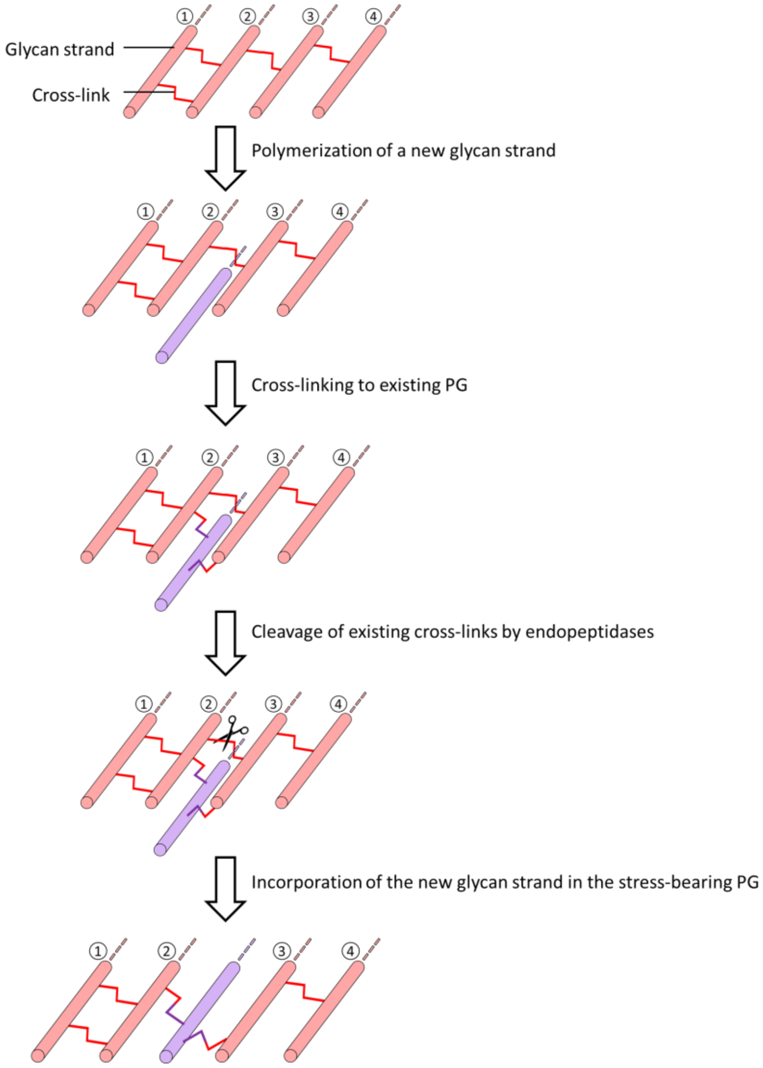

Supplementary Fig. 2. One-at-a-time mode of PG expansion in *E. coli*. According to the one-at-a-time model, expansion of the lateral wall involves the polymerization and cross-linking of the neo-synthesized chain beneath the existing PG mesh, followed by the cleavage of existing cross-links by endopeptidases. That second step leads to the introduction of the newly synthesized glycan strand into the stress-bearing mesh and the expansion of the surface of the side wall at a constant diameter. This sequential make-before-break mechanism enables the formation of the cross-links sheltered from the turgor pressure of the cytoplasm and eliminates the risk of breaching of the osmoprotective PG by endopeptidases. Color code: red, heavy isotopes; purple, light isotopes.

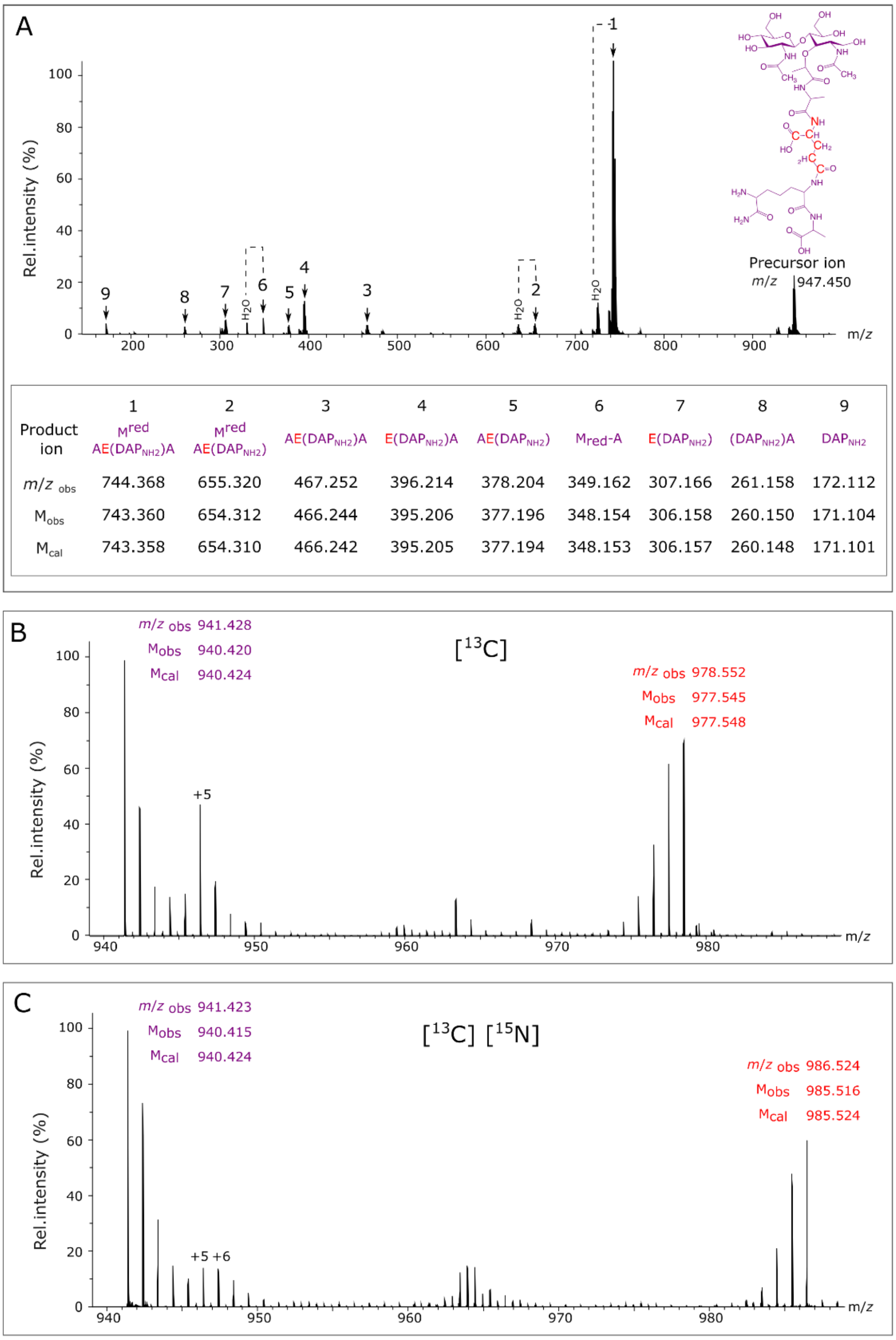

	1	2	3	4	5	6	7	8	9
Product ion	M^{red} AE(DAP_{NH2})A	M^{red} AE(DAP_{NH2})	AE(DAP_{NH2})A	E(DAP_{NH2})A	AE(DAP_{NH2})	M^{red}-A	E(DAP_{NH2})	(DAP_{NH2})A	DAP_{NH2}
m/z obs	744.368	655.320	467.252	396.214	378.204	349.162	307.166	261.158	172.112
M_{obs}	743.360	654.312	466.244	395.206	377.196	348.154	306.158	260.150	171.104
M_{cal}	743.358	654.310	466.242	395.205	377.194	348.153	306.157	260.148	171.101

Supplementary Fig. 3. Mass spectrometry analysis of hybrid isotopologues of the Tetra NH_2 muropeptide from *B. subtilis* strain 168. (**A**) Tandem mass spectrum of the +6 hybrid isotopologue. Precursor ion [GM^{red}-AE(DAP_{NH2})A], *m/z* 947.450; M_{obs} = 946.443; M_{cal} = 946.437. The fragmentation pattern indicates that the six heavy nuclei of the +6 isotopologue are all present in the D-Glu residue. The same conclusion was reached for the fragmentation of the +6 isotopologue of the disaccharide dipeptide (Supplementary Fig. 4). The +5 mass increment should correspond to the presence of five heavy nuclei and one light nucleus. Since the position of that light nucleus cannot be determined by tandem mass spectrometry, we analyzed the isotopic composition of the disaccharide-tetrapeptide following PG labeling with [^{13}C] only. Comparison of the mass spectra of the Tetra NH_2 muropeptide purified from PG labeled with both the [^{13}C] and [^{15}N] heavy isotopes (**B**) or with [^{13}C] only (**C**) shows that the five heavy nuclei of the +5 isotopologue correspond to the 5 carbon atoms of the D-Glu residue. Color code: red, heavy isotopes; purple, light isotopes. Abbreviations: M^{red}, reduced MurNAc; A, L-Ala or D-Ala; E, D-Glu; DAP_{NH2}, amidated diaminopimelic acid.

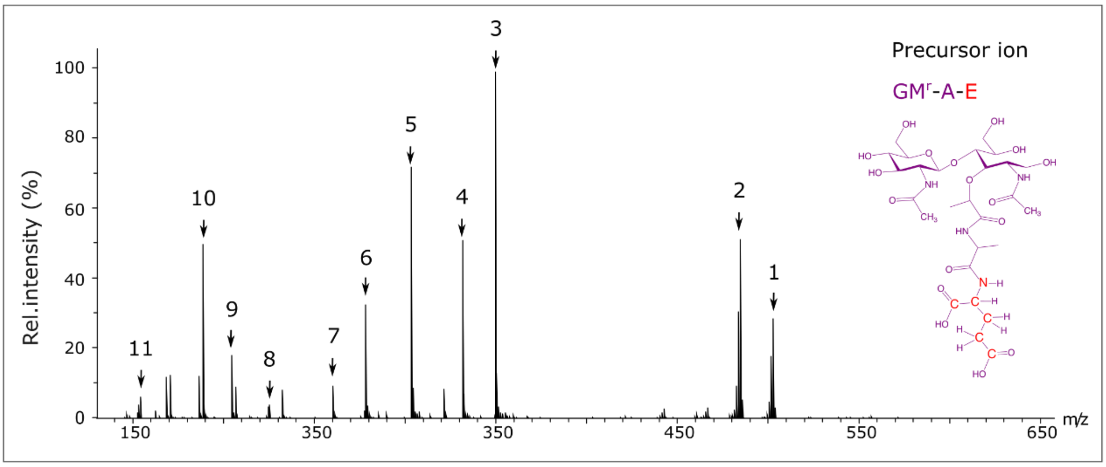

Product ion	1	2	3	4	5	6	7	8	9	10	11
	M^r-AE	1- H_2O	M^r-A	3 - H_2O	3 - CH_2O_2	M^r	6 - H_2O	AE	G	ND	E
m/z_{obs}	502.228	484.217	349.158	331.149	303.154	278.123	260.113	225.112	204.087	188.092	154.075
M_{obs}	501.218	483.207	348.150	330.142	302.146	277.114	259.105	224.103	203.079	187.075	153.067
M_{cal}	501.220	483.210	348.153	330.140	302.148	277.116	259.106	224.104	203.079	ND	153.067

Supplementary Fig. 4. Mass spectrometry analysis of hybrid isotopologues of the muropeptide GlcNAc-MurNAc-L-Ala-D-Glu from *B. subtilis* strain 168. Tandem mass spectrum of the +6 hybrid isotopologue. Precursor ion [GM^{red}-AE], *m/z* 705.303; M_{cal} = 704.300; M_{obs} = 704.295. The fragmentation pattern indicates that the six heavy nuclei of the +6 isotopologue are all present in the D-Glu residue. Color code: red, heavy isotopes; purple, light isotopes. Abbreviations: M^{red}, reduced MurNAc; A, L-Ala; E, D-Glu.

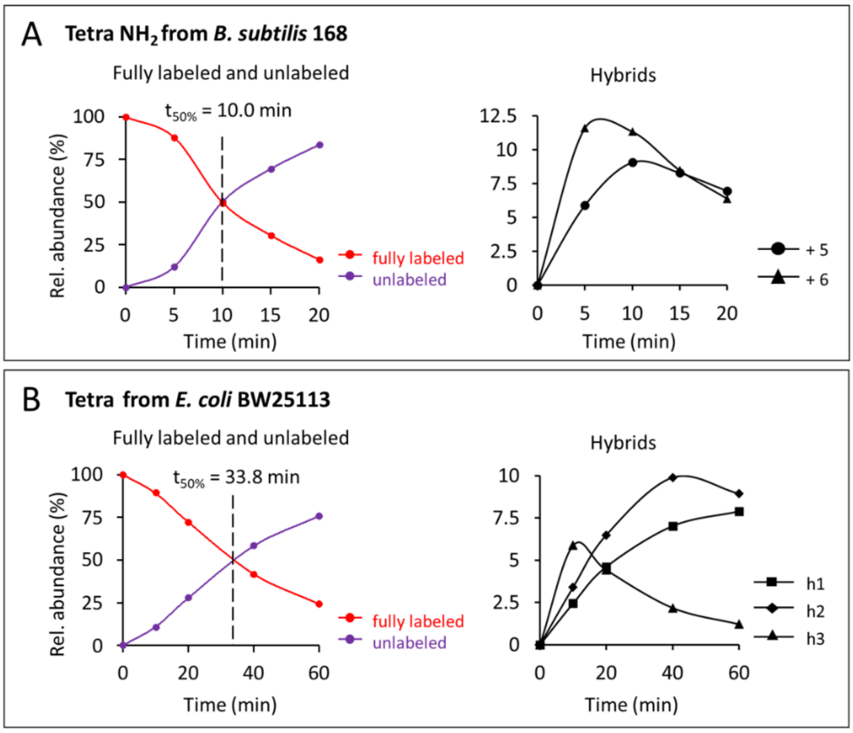

Supplementary Fig. 5. Kinetic analysis of the relative abundance of the Tetra NH_2 and Tetra muropeptides from *B. subtilis* strain 168 (A) and *E. coli* strain BW25113 (B). Left panels, relative abundance (%) of labeled *versus* unlabeled isotopologues. Right panels, abundance of hybrid isotopologues relative to all the isotopologues. The $t_{50\%}$ value is determined as the time at which the red and purple curves intersect. Data are representative of one out of four experiments (n=4). The h3 hybrid isotopologue contains light GlcNAc and heavy MurNAc-peptide moieties (Fig. 1B) and originates from the abundant cytoplasmic pool of the UDP-MurNAc-pentapeptide PG precursor[9]. Kinetic analyses show that the proportion of the h3 hybrid isotopologue levels off more rapidly than the proportions of hybrid isotopologues h1 and h2 containing recycled PG moieties. This difference was proposed to result from the quicker depletion of the pool of heavy UDP-MurNAc-pentapeptide in comparison to the decrease in labeled sugar and peptide moieties in the existing PG[9]. The kinetics observed for the hybrid isotopologues showing +5 and +6 mass increments might similarly be accounted for by the depletion of a preexisting pool of glutamate and 2-oxoglutarate, rather than the selective recycling of D-Glu from the existing PG.

Supplementary Table 1. Relative abundance (%) of light (unlabeled) and heavy (labeled) isotopologues of muropeptide monomers

Time (min)	Muropeptide									
	Tri$_{NH2}$		Tetra$_{NH2}$		Penta$_{NH2}$		Tri		Tri$_{NH2}$ deAc	
	Light	Heavy	Light	Heavy	Light	Heavy	Light	Heavy	Light	Heavy
**Biological replicate 1**										
0	0	100	0	100	0	100	0	100	0	100
5	18.6	81.4	12.0	88.0	13.5	86.5	15.5	84.5	13.6	86.4
10	55.7	44.3	50.3	49.7	50.5	49.5	46.3	53.7	49.1	50.9
15	77.4	22.6	69.6	30.4	70.7	29.3	75.8	24.2	67.8	32.2
20	87.2	12.8	83.8	16.2	84.9	15.1	82.5	17.5	82.5	17.5
**Biological replicate 2**										
0	0	100	0	100	0	100	0	100	0	100
5	8.2	91.8	6.6	93.4	6.6	93.4	7.4	92.6	4.4	95.6
10	49.3	50.7	41.6	58.4	42.5	57.5	47.9	52.1	36.0	64.0
15	72.6	27.4	67.6	32.4	67.1	32.9	69.7	30.3	59.0	41.0
20	84.5	15.5	81.5	18.5	82.1	17.9	82.9	17.1	71.7	28.3
**Biological replicate 3**										
0	0	100	0	100	0	100	0	100	0	100
5	11.9	88.1	7.5	92.5	9.3	90.7	17.7	82.3	10.7	89.3
10	37.4	62.6	29.0	71.0	33.3	66.7	39.1	60.9	33.2	66.8
15	56.4	43.6	49.7	50.3	56.7	43.3	54.7	45.3	45.2	54.8
20	71.6	28.4	64.2	35.8	72.4	27.6	65.9	34.1	62.8	37.2
**Biological replicate 4**										
0	0	100	0	100	0	100	0	100	0	100
5	7.0	93.0	4.0	96.0	6.2	93.8	7.7	92.3	9.5	90.5
10	29.6	70.4	20.9	79.1	25.9	74.1	23.4	76.6	32.5	67.5
15	51.3	48.7	41.4	58.6	49.7	50.3	51.0	49.0	51.8	48.2
20	65.6	34.4	55.7	44.3	60.5	39.5	61.7	38.3	61.5	38.5
**Mean**										
0	0	100	0	100	0	100	0	100	0	100
5	11.4	88.6	7.5	92.5	8.9	91.1	12.1	87.9	9.6	90.4
10	43.0	57.0	35.5	64.5	38.0	62.0	39.2	60.8	37.7	62.3
15	64.4	35.6	57.1	42.9	61.0	39.0	62.8	37.2	56.0	44.0
20	77.2	22.8	71.3	28.7	75.0	25.0	73.2	26.7	69.6	30.4
**Standard deviation**										
0	0	0	0	0	0	0	0	0	0	0
5	5.2	5.2	3.3	3.3	3.4	3.4	5.3	5.3	3.8	3.8
10	11.7	11.7	13.1	13.1	10.7	10.7	11.2	11.2	7.7	7.7
15	12.5	12.5	13.8	13.8	9.6	9.6	11.9	11.9	9.7	9.7
20	10.3	10.3	13.6	13.6	11.0	11.0	11.1	11.1	9.7	9.7

Abbreviation: Tri, tripeptide; Tetra, tetrapeptide; Penta, pentapeptide; NH_2, amidation of DAP; deAc, deacetylation of GlcNAc (n=4).

Supplementary Table 2. Relative abundance (%) of light (unlabeled) and heavy (labeled) isotopologues of muropeptide dimers

Time (min)	Muropeptide									
	Tetra→Tri NH_2*2		Tetra→Tri NH_2*1		Tetra→Tri NH_2*2 deAc*1		Tetra→Tetra NH_2*2		Tetra→Penta NH_2*2	
	Light	Heavy	Light	Heavy	Light	Heavy	Light	Heavy	Light	Heavy
**Biological replicate 1**										
0	0	100	0	100	0	100	0	100	0	100
5	2.1	97.9	6.9	93.1	2.3	97.7	0	100	0	100
10	34.3	65.7	46.5	53.5	31.5	68.5	27.8	72.2	42.8	57.2
15	63.0	37.0	77.0	23.0	65.6	34.4	60.2	39.8	72.4	72.4
20	85.7	14.3	87.2	12.8	79.9	20.1	76.8	23.2	83.7	83.7
**Biological replicate 2**										
0	0	100	0	100	0	100	0	100	0	100
5	0.8	99.2	2.6	97.4	0.8	99.2	0	100	0	100
10	25.0	75.0	30.6	69.4	15.2	84.8	16.6	83.4	46.8	53.2
15	59.1	40.9	68.1	31.9	47.0	53.0	46.1	53.9	63.7	36.3
20	80.7	19.3	83.8	16.2	67.0	33.0	59.7	40.3	76.6	23.4
**Biological replicate 3**										
0	0	100	0	100	0	100	0	100	0	100
5	1.0	99.0	2.4	97.6	4.8	95.2	0	100	0	100
10	13.5	86.5	19.2	80.8	11.3	88.7	8.3	91.7	18.1	81.9
15	39.6	60.4	46.0	54.0	30.5	69.5	23.9	76.1	41.7	58.3
20	59.0	41.0	62.8	37.2	47.7	52.3	40.9	59.1	61.2	38.8
**Biological replicate 4**										
0	0	100	0	100	0	100	0	100	0	100
5	0.4	99.6	1.2	98.8	1.1	98.9	0	100	0	100
10	7.9	92.1	11.4	88.6	9.1	90.9	4.8	95.2	14.2	85.8
15	25.7	74.3	38.9	61.1	29.0	71.0	17.2	82.8	33.1	66.9
20	44.5	55.5	57.1	42.9	48.3	51.7	34.2	65.8	51.6	48.4
**Mean**										
0	0	100	0	100	0	100	0	100	0	100
5	1.1	98.9	3.3	96.7	2.3	97.7	0	100	0	100
10	20.2	79.8	26.9	73.1	16.8	83.2	14.4	85.6	30.5	69.5
15	46.8	53.2	57.5	42.5	43.0	57.0	36.8	63.2	52.7	47.3
20	67.5	32.5	72.7	27.3	60.7	39.3	52.9	47.1	68.3	31.7
**Standard deviation**										
0	0	0	0	0	0	0	0	0	0	0
5	0.7	0.7	2.5	2.5	1.8	1.8	0	0	0	0
10	11.8	11.8	15.3	15.3	10.1	10.1	10.2	10.2	16.7	16.7
15	17.4	17.4	18.0	18.0	17.1	17.1	19.9	19.9	18.4	18.4
20	19.2	19.2	15.0	15.0	15.6	15.6	19.2	19.2	14.5	14.5

Abbreviation: Tri, tripeptide; Tetra, tetrapeptide; Penta, pentapeptide; NH_2, amidation of DAP; deAc, deacetylation of GlcNAc (n=4).

Supplementary Table 3. Delays between the synthesis of dimers and monomers

Monomer [a]	Dimer [b]	Replicate[c]	Difference in $t_{50\%}$ dimer – monomer [d]
Tri NH_2	Tetra→Tri $(NH_2)_2$	1	3.51
Tri NH_2	Tetra→Tri $(NH_2)_2$	2	3.52
Tri NH_2	Tetra→Tri $(NH_2)_2$	3	4.36
Tri NH_2	Tetra→Tri $(NH_2)_2$	4	6.76
Tetra NH_2	Tetra→Tetra $(NH_2)_2$	1	3.46
Tetra NH_2	Tetra→Tetra $(NH_2)_2$	2	4.83
Tetra NH_2	Tetra→Tetra $(NH_2)_2$	3	7.59
Tetra NH_2	Tetra→Tetra $(NH_2)_2$	4	7.52
Penta NH_2	Tetra→Penta $(NH_2)_2$	1	1.29
Penta NH_2	Tetra→Penta $(NH_2)_2$	2	-0.57
Penta NH_2	Tetra→Penta $(NH_2)_2$	3	3.55
Penta NH_2	Tetra→Penta $(NH_2)_2$	4	3.45
Tri NH_2 deAc	Tetra→Tri $(NH_2)_2$ deAc	1	2.47
Tri NH_2 deAc	Tetra→Tri $(NH_2)_2$ deAc	2	2.71
Tri NH_2 deAc	Tetra→Tri $(NH_2)_2$ deAc	3	3.69
Tri NH_2 deAc	Tetra→Tri $(NH_2)_2$ deAc	4	5.90
Tri	Tetra→Tri $(NH_2)_1$	1	-0.05
Tri	Tetra→Tri $(NH_2)_1$	2	2.10
Tri	Tetra→Tri $(NH_2)_1$	3	2.26
Tri	Tetra→Tri $(NH_2)_1$	4	2.19
Average ± standard deviation			3.53 ± 2.21

[a] The monomers contained amidated DAP (NH_2) and tripeptide, tetrapeptide, or pentapeptide stems (Tri NH_2, Tetra NH_2, Penta NH_2, respectively). Muropeptides with a tripeptide stem also contained glucosamine instead of *N*-acetylglucosamine (Tri NH_2 deAc) or DAP instead of amidated DAP (Tri).

[b] The dimers contained a tetrapeptide stem in the donor position and a tripeptide, tetrapeptide, or pentapeptide stem at the acceptor position [Tetra→Tri $(NH_2)_2$, Tetra→Tetra $(NH_2)_2$, Tetra→Penta$(NH_2)_2$, respectively]. Both DAP residues of these dimers were amidated. Dimers containing a tripeptide stem in the acceptor position also contained a deacetylated GlcNAc residue [Tetra→Tri $(NH_2)_2$ deAc] or one instead of two $(NH_2)_2$ DAP residues [Tetra→Tri $(NH_2)_1$]. The non-amidated DAP and deacetylated GlcNAc residues were not assigned to the donor or acceptor position of the dimers [$(NH_2)_1$ notation].

[c] For each one of four biological replicates (n=4), designated 1 to 4, monomers were matched to specific dimers according to the presence of the same stem in the monomer and in the acceptor position of the dimer [*i.e*. Tri NH_2 with Tetra→Tri $(NH_2)_2$, Tetra NH_2 with Tetra→Tetra $(NH_2)_2$, Penta NH_2 with Tetra→Penta $(NH_2)_2$]. Monomers and dimers containing a deacetylated GlcNAc were also matched [*i.e*. Tri NH_2 deAc with Tetra→Tri $(NH_2)_2$ deAc] as monomers containing a non-amidated DAP residue [*i.e*. Tri with Tetra→Tri $(NH_2)_1$].

[d] The delay between the synthesis of matched dimers and monomers was estimated by calculating the difference between the $t_{50\%}$ values. These data are those appearing in Fig. 3B.

Supplementary Table 4. Relative abundance (%) of light (unlabeled) and heavy (labeled) isotopologues of anhydro muropeptide

Time (min)	Muropeptide			
	Tri NH_2 Anh		Tetra NH_2➔Tri NH_2 Anh	
	Light	Heavy	Light	Heavy
**Biological replicate 1**				
0	0	100	0	100
5	9.9	90.1	1.7	98.3
10	36.1	63.9	31.2	68.8
15	57.9	42.1	66.3	33.7
20	72.7	27.3	79.9	20.1
**Biological replicate 2**				
0	0	100	0	100
5	6.4	93.6	0	100
10	34.5	65.5	18.4	81.6
15	55.4	44.6	46.4	53.6
20	72.3	27.7	74.1	25.9
**Biological replicate 3**				
0	0	100	0	100
5	8.1	91.9	0.7	99.3
10	26.3	73.7	10.0	90.0
15	43.3	56.7	29.6	70.4
20	55.6	44.4	46.4	53.6
**Biological replicate 4**				
0	0	100	0	100
5	5.5	94.5	0.3	99.7
10	25.6	74.4	5.4	94.6
15	39.7	60.3	22.7	77.3
20	50.2	49.8	39.2	60.8
**Mean**				
0	0	100	0	100
5	7.5	92.5	0.7	99.3
10	30.6	69.4	16.2	83.8
15	49.0	51.0	41.3	58.7
20	62.7	37.3	59.9	40.1
**Standard deviation**				
0	0	0	0	0
5	2.0	2.0	0.7	0.7
10	5.5	5.5	11.3	11.3
15	8.9	8.9	19.5	19.5
20	11.5	11.5	20.1	20.1

Abbreviation: Tri, tripeptide; Tetra, tetrapeptide; NH_2, amidation of DAP; Anh, anhydro (n=4).
