# Supplementary material for: Mechanism of lateral cell-wall expansion at a constant diameter in Bacillus subtilis
Source: Nat Commun. 2025 Jul 19;16:6671. doi: 10.1038/s41467-025-61900-0 (PMC12276332; doi:10.1038/s41467-025-61900-0)
Supplement: Supplementary file 2 — Description of Additional Supplementary Files [file 41467_2025_61900_MOESM2_ESM.pdf]

Description of Additional Supplementary Files:

Supplementary Data 1: Spread sheet for the simulation of the replacement of heavy by light PG as a function of the generation time and PG turnover.

The spread sheet contains:

Column A & B: the generation time in minutes (line 1); the total amount of PG at t=0 min (arbitrary unit) (line 2); the time increment used to generate time points in column C (line 3); the turnover constant in minute (line 4); the value of the Ln(2) (line 20); the deduced half-life of the PG (line 21).

Column C: Time scale used for the simulation

Column D: Kinetics of the total amount of PG (arbitrary unit)

Column E: Kinetics of the increase of the PG amount (arbitrary unit)

Column F: Degradation of PG per time increment (arbitrary unit)

Column G: Total PG degradation (arbitrary unit)

Column H: Amount of neosynthesized PG (arbitrary unit)

Column I: Amount of existing PG (arbitrary unit)

Column J: Percentage of heavy PG

Column K: Percentage of light PG

The values in column J and K are used to generate the graph. Each cell of the spread sheet contains the formula used to generate the variations in the parameter defined in column C to K.

Supplementary Data 2: Spread sheets of original mass spectrometry data for the 14 most abundant muropeptides used for kinetic analyses.

Each sheet in this file holds MS data for one of the 14 most abundant muropeptide used for kinetic analyses. The first row of each sheet indicates the rpHPLC fraction containing the muropeptide and the biological replicate it comes from.

Column A. Incubation time after the medium switch.

Column B. Observed *m*/*z* value

Column C. Observed monoisotopic mass

Column D. Calculated monoisotopic mass

Column E. Intensity for the ion with the indicated *m*/*z* value (arbitrary units)

Column F. Difference between the observed and calculated monoisotopic masses in part per million (ppm)

Column G. Deduced structure. A, L-Ala or D-Ala; E, γ-D-glutamic acid; DAP, diaminopimelic acid; NH2, amidation of DAP; deAc, deacetylation of GlcNAc; →, 4→3 cross-link; +5.01 and +6.01, mass increments due to the presence of 5 or 6 heavy nuclei at the 2nd position of stem peptides. The presence of light (newly synthesized) and heavy (existing) moieties of muropeptides is indicated in purple and red, respectively.

Supplementary Data 3: Synthetic route for the obtention of the ethinyl-D-Ala-D-Ala compound and NMR spectra.
