# Supplementary material for: Mechanism of lateral cell-wall expansion at a constant diameter in Bacillus subtilis
Source: Nat Commun. 2025 Jul 19;16:6671. doi: 10.1038/s41467-025-61900-0 (PMC12276332; doi:10.1038/s41467-025-61900-0)
Supplement: Supplementary file 5 — supplementary data 3 [file 41467_2025_61900_MOESM5_ESM.docx]

**Supplementary Data 3. Synthesis of ethinyl-D-Ala-D-Ala.**

All starting materials were obtained from commercial sources and were used without further purification. NMR spectra were acquired on a Bruker Advance 400 MHz for 1H-NMR experiments and 100 MHz for 13C-NMR experiments. Chemical shifts are reported in ppm (*δ*) relative to the solvents: 1H *δ*(CD3OD) = 3.3 ppm, 13C *δ*(CD3OD) = 49.15 ppm. Accurate mass spectra were recorded on a time-of-flight (TOF) spectrometer (Waters, XEVO G2-S qTOF) and on an LTQ Orbitrap XL spectrometer with electrospray ionization (ESI, Thermo Scientific).

**Scheme 2.** Synthesis of ((*R*)-2-amino-3-alkynepropanoyl)-D-alanine (ethinyl-D-Ala-D-Ala, compound **4**)

**2-(Trimethylsilyl)ethyl (*tert*-butoxycarbonyl)-D-alaninate (compound 1)**

Compound **1** was obtained according to Van Nieuwenhze’s process (G. W. Liechti, E. Kuru, A. Kalinda, Y. V. Brun, M. Van Nieuwenhze, A. T. Maurelli, *Nature* **2014**, *506*, 507-510).

**2-(Trimethylsilyl)ethyl((*R*)-3-alkyne-2-((*tert*-butoxycarbonyl)amino)propanoyl)-D-alaninate (compound 2)**

Compound **1** (246.6 mg, 0.85 mmol) was diluted in a solution of HCl in dioxane (4 N, 4 mL) and the mixture was stirred for 2 h at room temperature. The solvent was removed under vacuum and the compound was allowed to dry under high vacuum (oil pump) for 4 h and the deprotected product was used as such for the next step. This compound and compound **2** (182 mg, 0.85 mmol) were dissolved in a 1:1 mixture of dry CH_2_Cl_2_/DMF (10 mL). HATU (356 mg, 0.94 mmol) and Hünig’s base (242 mg, 1.87 mmol, diluted in 0.6 mL of CH_2_Cl_2_) were successively added and the mixture was stirred overnight at room temperature. The solvent was then removed, and ethyl acetate was added. After it was washed with an aqueous solution of 10% citric acid followed by 5% NaHCO_3_, the organic layer was dried over MgSO_4_ and evaporated. After flash chromatography (silica gel, 15% ethyl acetate/cyclohexane), the product **3** was isolated as a colorless oil (203 mg, 62%); ^1^H NMR (400 MHz, CDCl_3_) *δ* (ppm) 0.05 (s, 9H), 1.02 (m, 2H), 1.41 (d, *J* = 7.1 Hz, 3H), 1.47 (s, 9H), 2.09 (t, *J* = 2.6 Hz, 1H), 2.61 (ddd, *J* = 16.8, 6.4, 2.6 Hz, 1H), 2.79 (ddd, *J* = 16.8, 5.6, 2.6 Hz, 1H), 4.24 (m, 2H), 4.33 (m, 1H), 4.54 (m, 1H); ^13^C NMR (100 MHz, CDCl_3_) *δ* (ppm) 1.4 (3×CH_3_), 17.5 (CH_2_), 18.6 (CH_3_), 22.6 (CH_2_), 28.4 (3×CH_3_), 48.6 (CH), 52.9 (CH), 64.1 (CH_2_), 71.8 (CH), 79.5 (C), 80.6 (C), 155.5 (C), 169.8 (C), 172.8 (C); HRMS (ESI+) m/z calc. for C_18_H_33_O_5_N_2_Si [M+NH]^+^ 385.2153, found 385.2151.

^1^H NMR spectrum (400 MHz, CDCl_3_)

^13^C NMR spectrum (100 MHz, CDCl_3_)

**((*R*)-2-amino-3-alkyne-propanoyl)-D-alanine (ethinyl-D-Ala-D-Ala, compound 4).**

Compound **3** (141.7 mg, 0.37 mmol) was diluted in TFA (3 mL) in an ice bath and the mixture was stirred for 4 h at room temperature. The solvent was removed under vacuum and the product was dried to dry under high vacuum (oil pump) for 4 h. Dry ether (10 mL) was added, and the mixture was stirred overnight under argon. The white solid was obtained after filtration and drying under high vacuum, compound **4** was obtained as white solid (85 mg, 74 %); ^1^H NMR (400 MHz, CD_3_OD) *δ* (ppm) 1.41 (d, *J* = 7.3 Hz, 3H), 2.60 (t, *J* = 2.7 Hz, 1H), 2.77 (ddd, *J* = 17.5, 7.8, 2.7 Hz, 1H), 2.91 (ddd, *J* = 17.5, 5.2, 2.7 Hz, 1H), 4.05 (dd, *J* = 7.8, 5.2 Hz, 1H), 4.40 (q, *J* = 7.3 Hz, 1H); ^13^C NMR (100 MHz, CD_3_OD) *δ* (ppm) 17.9 (CH_3_), 22.6 (CH_2_), 50.2 (CH), 52.9 (CH), 75.1 (CH), 77.5 (C), 118.2 (q, *J* = 292.0 Hz, CF_3_), 163.4 (q, *J* = 35.0 Hz, C), 168.6 (C), 176.3 (C); HRMS (ESI+) m/z calc. for C_8_H_13_N_2_O_3_ [M]^+^ 185.0921, found 185.0920.

^1^H NMR spectrum (400 MHz, CD_3_OD)

^13^C NMR spectrum (100 MHz, CD_3_OD)
